# Depth-Resolved Deformation Monitoring and Short-Term Forecasting with Multipoint Extensometers in Hydraulic Tunnels

**DOI:** 10.3390/s26216801

**Published:** 2026-10-28

**Authors:** Jintao Liao, Siyu Chen, Kai Zhou, Xin Chen, Xiantao Hu, Yifan Wang, Langhui Wu, Chang Chen, Qingfeng Lv

**Affiliations:** 1 POWERCHINA Zhongnan Engineering Corporation Limited, Changsha 410014, China; l1308802837@outlook.com (J.L.); csychangshaligong@163.com (S.C.); zk19940511@gmail.com (K.Z.); 15111244547@163.com (X.C.); 15042206500@163.com (X.H.); wwwyf_csu@126.com (Y.W.); huiwu2008@126.com (L.W.); 18874209552@163.com (C.C.); 2 School of Civil Engineering and Mechanics, Lanzhou University, Lanzhou 730000, China

**Keywords:** multipoint extensometer, deformation monitoring, adjacent-anchor segment, hydraulic tunnel, irregular sampling, residual forecasting, prediction interval

## Abstract

**Highlights:**

**What are the main findings?**

- Source-linked readings from 65 channels yielded 45 adjacent-anchor depth intervals with actual observation times retained.
- On 84 later forecasts, direct correction had the lowest point error; the gated variant improved persistence mean absolute error (MAE) by 4.59% and narrowed empirical intervals at the same observed coverage.

**What are the implications of the main findings?**

- Readout checks and adjacent-anchor states locate records and depth intervals that warrant engineering review.
- The next-reading forecast provides a local comparison for follow-up measurements; its correction and interval require validation at each instrument.

**Abstract:**

Multipoint extensometers measure rock movement at several depths, but channel plots can obscure changes between anchors. We linked exported readouts to conversion coefficients, formed adjacent-anchor states and forecast the next reading using actual observation intervals. Records from a pumped-storage water-conveyance system comprised 20 instruments, 65 channels, 45 segments, 2898 segment observations and 2667 forecast samples. Numerical reconstruction matched 4209 of 4211 displayed displacements within 10^−6^ mm; it did not independently calibrate the instruments. We assessed 84 seven-day forecasts on two later monitoring dates. Direct ExtraTrees residual correction gave the lowest mean absolute error (MAE), 0.024819 mm. A validation-selected magnitude gate changed 13 forecasts and reduced MAE from the persistence value of 0.026113 to 0.024915 mm (4.59%). A lagged linear comparator gave 0.026001 mm. The gated nominal 95% interval covered 82/84 observations and was 10.38% narrower than the persistence interval at the same observed coverage; common-calendar calibration covered only 77/84. Instrument-held-out refitting improved 2 of 19 instruments. Source-linked segment states support depth-specific record review. The small forecast gain should be treated as a local analytical result until calibration, independent monitoring rounds and an instrument-level uncertainty budget are available.

## 1. Introduction

Multipoint extensometers provide repeated measurements of rock movement at selected borehole depths. Field studies have used them to examine internal tunnel deformation alongside excavation and support records [1,2,3]. In routine monitoring, however, readings are commonly exported as separate displacement columns. These show movement at each anchor but do not directly identify the depth interval in which relative movement accumulated. This matters when an engineer must decide which part of a borehole to inspect after a change.

A routine export may contain sensor readouts and converted displacement while conversion coefficients, anchor geometry and instrument specifications are stored separately. Plotting each displacement column independently leaves this measurement chain unchecked. It also makes a change shared by neighbouring anchors difficult to separate from a change concentrated between them. Differential readings between fixed points are established in extensometry [1,4]. Our contribution is their source-linked implementation across instruments with different anchor counts, followed by forecasting and auditing of the resulting depth-specific states at actual observation times.

Tunnel-deformation forecasts have used multivariate adaptive regression splines, neural networks, recurrent networks and hybrid models [5,6,7]. Graph-attention and long short-term memory (LSTM)–random-forest studies address spatial or multidepth relationships [8,9]. Those methods do not remove the need to define the measured target and verify its source. For exported extensometer records, three questions remain practical, namely whether the displacement conversion can be checked, which adjacent depth interval is changing, and whether a forecast improves on the last observation.

Monitoring intervals vary with access and field schedules. A fixed-size sequence of displacement values therefore contains unequal periods between readings. Methods used with irregular time series retain elapsed time explicitly [10,11]. Here, each forecast uses five valid observations and their timestamps. The target is the next measured state of a specified anchor interval. Recent rates and contemporaneous states of other intervals on the same instrument are calculated at the forecast origin.

Persistence is an essential reference for slowly changing measurements. A forecast of cumulative displacement can have a high coefficient of determination while providing little improvement on the last reading. Chronological testing, explicit baselines and increment-sensitive error measures are therefore needed [12,13,14,15]. Model selection and interval calibration also require separate records [16]. These considerations lead to a residual forecast; the model estimates a correction to the current segment state and a validation-selected gate restricts when that correction is applied.

Prediction intervals provide a range for the next observed state and complement the point forecast [17,18,19,20]. Adjacent segments share an anchor, so their errors are dependent. The measurement conversion and the prediction procedure must preserve this dependence. We assess source-readout perturbations at shared anchors and compare intervals calibrated on identical records. Measurement uncertainty is considered separately through the propagation of adjacent-channel errors [21].

We ask whether source-linked adjacent-anchor states improve the review of depth-specific records and whether a short-term model provides information beyond persistence. The study covers four areas of one pumped-storage water-conveyance system. Two weekly monitoring rounds after the development cutoff provide later-date tests. The analysis follows source readout, conversion, segment construction, time-aware features, point prediction and empirical interval assessment. A held-out-instrument analysis examines transfer beyond the instruments used for fitting.

The study evaluates three connected aspects of this workflow:

1. Reconstruction and metadata reconciliation identify numerical and instrument-record inconsistencies before adjacent-anchor states are calculated.
2. A common segment representation accommodates three- and four-anchor instruments and preserves depth, elapsed time and shared-anchor dependence.
3. Matched comparisons with persistence, simple statistical baselines and direct residual correction test the incremental value of the forecasting step. Sensitivity analyses examine gate settings, history length, duplicate records and chronological partitioning.

## 2. Materials and Methods

### 2.1. Engineering Background and Monitoring Records

**Figure 1:**
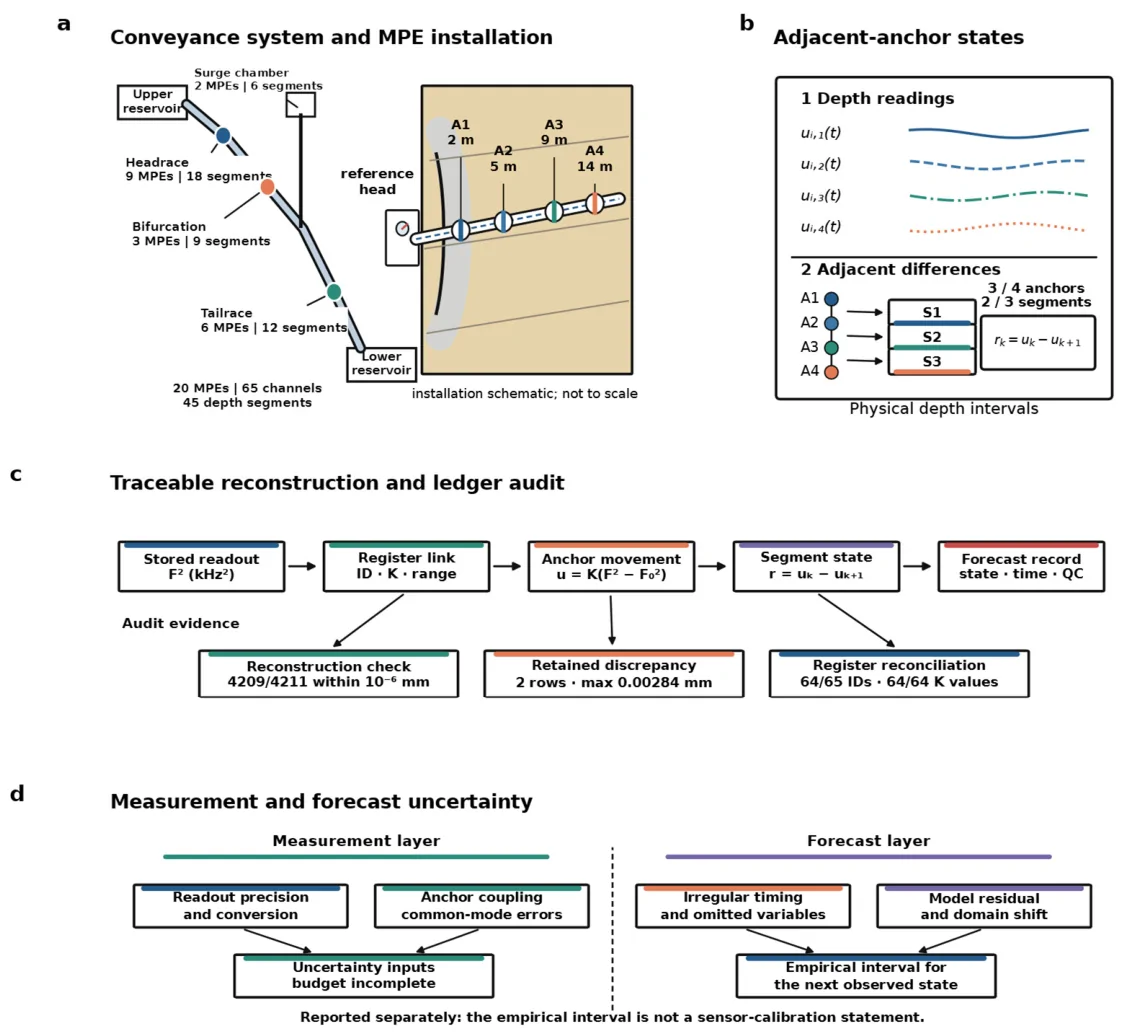
Measurement definition and audit boundary for multipoint extensometers (MPEs). (**a**) Author-drawn vector schematic using the recorded 2, 5, 9 and 14 m anchor depths of M4b4-1. Geometry and rock texture are schematic. (**b**) Depth-specific channels and adjacent-anchor differences. The curves illustrate the operator and are not field observations. Three and four anchors yield two and three adjacent segments, respectively. (**c**) Source-linked reconstruction and register reconciliation. (**d**) Measurement uncertainty and forecast uncertainty require separate evidence. Computational provenance does not establish International System of Units (SI) traceability. In (**b**), line styles distinguish anchor channels and their adjacent segments. ID denotes sensor identifier and QC denotes quality control.

The study concerns the headrace tunnel, tailrace tunnel, headrace bifurcation and headrace surge chamber of a pumped-storage water-conveyance system. The four areas contained 9, 6, 3 and 2 retained physical multipoint extensometers (MPEs), respectively. Their 65 anchor channels defined 45 adjacent-anchor segments. Records span 16 November 2024 to 4 September 2026. We used 21 August 2026 as the retrospective development cutoff and reserved the 28 August and 4 September observations for testing. Figure 1 illustrates the measurement geometry, conversion and segment definition. Table 1 summarises the retained monitoring records.

The source package comprised monitoring workbooks, an instrument ledger and engineering-location records. The analysis retained area, physical instrument identifier, installation position, anchor index and depth, timestamp, sensor readout, conversion coefficient and displayed displacement. Chainage was retained where available. Twelve rows shared an instrument-anchor-timestamp key with another row, representing six duplicated pairs. Duplicate entries were averaged and flagged before segment construction. The original rows remained in the audit archive. Long gaps were not interpolated and no synthetic field readings were added.

The available geological records assigned the linked monitoring sections to rock Class III, leaving no between-class contrast for modelling. Timestamped excavation, support, groundwater, temperature and independent reference measurements were unavailable. Consequently, an observed change here cannot be attributed to construction rather than instrument drift or environmental response. The experimental target is the next measured segment state, not an independently verified physical displacement.

### 2.2. Readout Reconstruction and Instrument Reconciliation

The monitoring workbooks label the sensor readout *F*^2^ in kHz^2^ and the channel coefficient *K* in mm kHz^−2^. These labels and the workbook conversion convention are retained here. No independent verification of the readout scale or calibration certificate was available, so numerical reconstruction does not establish International System of Units (SI) traceability. For anchor *k* of instrument *i*, the reference epoch *t*_0_ was the first retained valid displacement record, and the reconstructed displacement was

(1) $$u_{i,k}\left(t_{m}\right)=K_{i,k}\left(F_{i,k}^{2}\left(t_{m}\right)-F_{i,k,0}^{2}\right).$$

The analysis retained the workbook displacement, reconstructed displacement and their residuals as separate audit fields. Reconstructed values were used for segment calculations. Of 4211 anchor observations, 4209 reproduced the displayed result within 10^−6^ mm; two differed by 0.00284 mm. This checks the arithmetic under the workbook convention. It does not verify the readout unit, coefficient calibration or physical movement (Figure 1c).

Sensor identifiers, *K* values, nominal model and range were then linked to the instrument ledger. Sixty-four of 65 anchor identifiers were matched. All 64 linked *K* values agreed exactly. Three linked channels had model and range disagreements. The workbook header identified NVJ-100 and 100 mm, whereas the ledger identified NVJ-150 and 150 mm. One identifier remained unlinked. No inferred correction was applied. A sensitivity analysis later excluded observations associated with these metadata discrepancies.

A worked example illustrates the reference definition. For M3b1-1, the 2 m and 5 m anchors share the 27 November 2025 retained baseline. Their baseline readouts were 3159.2 and 3462.0, with *K* = 0.0181 for both. Readouts on 4 September 2026 were 3165.8 and 3455.1. Equation (1) gives 0.11946 and −0.12489 mm, so their adjacent-segment difference is 0.24435 mm. This calculation uses the exported readout scale and does not infer absolute rock movement. Supplementary Table S2 records the inputs.

### 2.3. Definition of Adjacent-Anchor Deformation

Let *u_i_*_,*k*_(*t_m_*) denote the relative displacement recorded by anchor *k* of physical instrument *i* at observation time *t_m_*. Anchor depth *z* is measured from the borehole collar and increases towards the deeper anchor (Figure 1a). The relative deformation assigned to the interval between anchors *k* and *k* + 1 is

*r_i_*_,*k*_(*t_m_*) = *u_i_*_,*k*_(*t_m_*) − *u_i_*_,*k*+1_(*t_m_*). (2)

Positive *r* denotes a larger reconstructed displacement at the shallower anchor than at its deeper neighbour, relative to their common reference epoch. The physical extension/compression sign could not be independently checked against a manufacturer convention and is not assigned here. All 20 instruments had a common retained baseline epoch across their anchors. Differencing cancels a component that acts equally on both channels (Figure 1b), but anchor coupling, borehole orientation and discontinuities still affect interpretation. For unequal anchor spacing, the equivalent change per unit segment length was calculated as

(3) $$\varepsilon^{\mathrm{eq}}\left(t_{m}\right)=\frac{r_{i,k}\left(t_{m}\right)}{h_{i,k}}.$$

Here *h_i_*_,*k*_ = *z_i_*_,*k*+1_ − *z_i_*_,*k*_ > 0 is the segment thickness. The study reports *r* in mm and *h* in m, so the equivalent quantity is expressed in mm m^−1^; division by 1000 gives the corresponding dimensionless ratio. The forecast target was *r_i_*_,*k*_. The normalised quantity was used for interpretation and geometry checks, not as an independent predictor, because *r* and *h* already determine it.

At each timestamp, the total shallow-to-deep relative movement of the physical instrument and the mean, standard deviation and maximum absolute state across its available segments were calculated. These variables describe the contemporaneous instrument context using only readings at the historical-window endpoint.

### 2.4. Propagation of Adjacent-Channel Uncertainty

The segment state inherits uncertainty from both adjacent channels. Let *u*_c_(*u_i_*_,*k*_) and *u*_c_(*u_i_*_,*k*+1_) be their combined standard uncertainties and let *ρ_i_*_,*k*_ be the correlation coefficient between the channel errors at a common timestamp. Suppressing the instrument and segment indices, the first-order propagation for the difference in Equation (2) gives [21]

(4) $$\begin{array}{c} {u_{c}}^{2}\left(r\right)={{\;u}_{c}}^{2}\left(u_{k}\right)+{{\;u}_{c}}^{2}\left(u_{k+1}\right)\\ -2\rho u_{c}\left(u_{k}\right)u_{c}\left(u_{k+1}\right). \end{array}$$

Positive correlation can reduce the propagated uncertainty when common reference or environmental effects cancel in the adjacent-channel difference. Independent channel noise adds in quadrature. A fault at one anchor can affect the two segment states that share that anchor, which creates both statistical dependence and a recognisable diagnostic pattern. Temporal increments add a second layer, because readings at two epochs contribute to the change. Dividing by elapsed time transfers timestamp uncertainty and amplifies noise when the interval is short.

The exports provide stored readout precision but no complete calibration certificates, temperature-compensation histories, anchor-coupling tests or channel-covariance estimates. Equation (4) therefore defines the propagation relationship without assigning a numerical combined measurement uncertainty. The intervals in Section 2.8 are calibrated from forecast residuals and describe the next-observation prediction error. Table 2 summarises the measurement chain and audit boundaries.

### 2.5. Irregular Sampling and Forecast Samples

Each instrument-segment series was ordered by its recorded timestamp. A sample used the preceding five valid observations to predict the next valid observation, provided that the horizon was positive and no greater than 14 days. The task is therefore a next-observation forecast with an explicit natural-time horizon. It does not assume equal observation spacing. For deployment, the horizon can be set to the scheduled next reading. In the retained dataset, the mean horizon was 6.70 d and the median was 7 d.

For two consecutive retained observations, the elapsed interval was

Δ*t_m_* = *t_m_* − *t_m_*_−1_. (5)

All rate-like variables were normalised by their actual Δ*t*. Within the five-observation history, slopes were calculated for every pair using the corresponding elapsed time, and their median was used as a Theil-Sen-type robust trend. The ten pairwise calculations per window were computationally negligible. An acceleration proxy was obtained from the two latest time-normalised velocities divided by their mean elapsed interval. Table 3 lists the feature groups.

Each retained segment had at least 13 valid observations. Development targets dated on or before 21 August 2026 were divided separately within each instrument-segment series into chronological training (65%), validation (17.5%) and calibration (17.5%) blocks, yielding 1660, 449 and 474 samples. For each target, the five history timestamps preceded the target; the latest history reading was the forecast origin. Cross-segment summaries used only the other segments measured on that same instrument at that origin. No target-date reading entered a predictor. The test comprised 84 seven-day targets from 42 segments and 19 instruments, 42 on each later date. Development periods overlapped between instruments, so the primary test addresses later readings at known instruments. The common-calendar analysis in Section 2.9 checks this choice.

### 2.6. Persistence and Residual Forecasting

The primary reference forecast was persistence:

*r*^pers^(*t* + *τ*) = *r*(*t*). (6)

The last measured segment state is carried forward to the requested horizon. A robust-trend comparator extrapolated the median pairwise slope over the actual forecast horizon, without slope clipping. Persistence was the reference because most next increments were small relative to the accumulated segment state.

The learner estimated the increment beyond persistence. Ridge regression, random forest [22], histogram gradient boosting and ExtraTrees [23] were compared on the 449 validation samples. We also evaluated a low-dimensional lagged linear model and an exponentially weighted historical-velocity extrapolation. Their settings were selected on validation only, after fitting the linear coefficients on the training block. The selection objective was overall mean absolute error (MAE) plus one-half of high-change MAE. High change was defined by the pooled training-only 80th percentile of absolute increments. ExtraTrees with a minimum leaf size of 10 had the lowest objective among the full-feature learners (Table 4). Persistence had a lower validation objective than every fitted comparator, so the test assesses whether a correction adds value despite this conservative validation signal.

The selected model used 600 trees, at least 10 samples per leaf, 80% of features considered at each split and a random seed of 20260904. Numeric features were standardised. Area, installation position and segment index were one-hot encoded. Physical instrument identity was withheld to limit direct sensor memorisation.

### 2.7. Magnitude-Gated Residual Correction

A residual learner can add unnecessary corrections to a nearly stable sequence. The gated variant retained persistence when the absolute predicted increment was below a validation-selected threshold. Otherwise, it applied a shrunken correction. Let $\Delta \hat{r}$ denote the predicted increment for the requested segment and horizon. The gated forecast was

(7) $$r^{\mathrm{gate}}\left(t+\tau \right)=r\left(t\right)+I\left(\left|\Delta \hat{r}\right|\;\geq \;\gamma \right)\lambda \Delta \hat{r}.$$

Here, *γ* is the magnitude threshold, *λ* the shrinkage factor and I(·) the indicator function. Candidate *γ* values were quantiles of absolute validation predictions, and *λ* ranged from 0.1 to 1.0 in steps of 0.1. The validation objective was overall MAE plus one-half of the high-change MAE. High change meant a target increment of at least 0.024048 mm in absolute value, the pooled training-only 80th percentile. The selected *γ* was 0.014998 mm and *λ* was 0.9. They were fixed before the two later dates were scored. The gate activated on 13/84 test forecasts. A local perturbation of *γ* by ±20% and *λ* between 0.7 and 1.0 is reported in Table S5. This is a post hoc sensitivity check, not a second selection step.

### 2.8. Residual-Calibrated Prediction Intervals

Absolute residuals of the gated model were calculated only on the calibration block. Let *e_j_* = |*y_j_* − *ŷ_j_*| and let *e*_(1)_ ≤ ··· ≤ *e*_(nc)_ denote the ordered calibration residuals. For nominal coverage 1 − *α* = 0.95, define *ℓ* = min{*n*_c_, ⌈(*n*_c_ + 1)(1 − *α*)⌉}. The symbol *ℓ* is an order-statistic index and is distinct from the anchor-segment index *k*. The finite-sample residual quantile was

(8) $${\hat{q}}_{1-\alpha }=e_{\left(l\right)}.$$

The primary procedure pooled absolute calibration residuals across segments to obtain one global half-width. Area-specific quantiles were calculated as diagnostics. The pooled rule was applied identically to persistence, the direct residual model and the gated model in a matched interval comparison. Suppressing indices and time arguments, the interval was

(9) $${\mathrm{PI}}_{1-\alpha }=\left[\hat{r}-{\;\hat{q}}_{1-\alpha },\hat{r}+{\hat{q}}_{1-\alpha }\right].$$

For the gated model, the nominal 95% half-width was 0.10273 mm. Diagnostic area-specific half-widths were 0.10272, 0.15680, 0.03331 and 0.04981 mm for the headrace, tailrace, bifurcation and surge chamber. The 474 calibration samples determined these quantities before test scoring. Serial, within-instrument and shared-date dependence prevent an exact coverage guarantee [17,18,19,20]. Coverage is therefore reported as an empirical count on the later observations.

### 2.9. Evaluation and Sensitivity Analyses

Point forecasts were evaluated using MAE, root mean square error (RMSE) and bias, defined as the mean prediction minus observation. High-change results used the pooled training-only 80th percentile because numerous small increments can conceal errors during larger changes. Cumulative-state *R*^2^ was not used as the main performance claim. Interval comparisons used identical calibration and test records for all three methods. In addition to coverage and full width, the mean central interval score was reported. For bounds *L* and *U* and observation *y*, the score is

(10) $${\mathrm{IS}}_{\alpha }=\left(U-L\right)+\frac{2}{\alpha }\left[{\left(L-y\right)}_{+}+{\left(y-U\right)}_{+}\right],$$

where (*x*)_+_ = max(*x*, 0). The score has the same unit as the target and penalises both excessive width and missed observations. Lower values are preferable.

Paired absolute-error differences were summarised by physical instrument. Ten thousand bootstrap draws resampled the 19 test instruments as clusters and retained all observations from each sampled instrument. The 2.5th and 97.5th percentiles form a descriptive resampling interval for the sample-weighted mean difference. Adjacent segments and overlapping histories were not treated as independent replicates. Both test dates are shared across instruments, so instrument clustering alone cannot estimate date-to-date variation. We report per-date errors and make no population-level significance claim from this interval.

Leave-one-instrument-out (LOIO) refitting removed the held-out instrument from the records used to fit the learner and select the gate. Only its later test observations were scored, with aggregate errors weighted by sample count. The learner family and minimum leaf size retained their globally selected values. LOIO therefore provides a transfer diagnostic conditional on those settings. Prediction intervals were not evaluated in this diagnostic.

Readout sensitivity was evaluated at the source level. In each replicate, one uniform perturbation was assigned to every unique physical anchor and timestamp. The same perturbation was reused wherever that reading appeared in overlapping windows or adjacent segments. Baseline readings were perturbed once and retained as shared references across time. Anchor displacements, segment states, time-dependent features and cross-segment contexts were then reconstructed consistently. Persistence, direct residuals and gated forecasts used the same perturbed histories and targets, with fitted model parameters, gate and shrinkage held fixed. The primary half-step was ±0.05 in the workbook-reported kHz^2^ scale, corresponding to half the stored 0.1 step. Wider ±0.25 and ±0.50 levels were stress tests. There were 300 replicates per non-zero level. This sensitivity exercise does not include calibration, temperature or coupling uncertainty. A separate metadata sensitivity removed test samples associated with the unlinked identifier or three model/range discrepancies, without refitting.

A further retrospective sensitivity used a common calendar: training targets up to 30 April 2026, validation in May–June and calibration from 1 July to 21 August. These blocks contained 1860, 393 and 330 samples, with the same 84 test targets. Learner and gate selection used only the alternative training and validation blocks; the interval used its calibration block. We also rebuilt the full source-derived analysis with five, seven and nine observations per history, selecting the gate within each development partition. Finally, we replaced the duplicate-pair mean rule with first-entry and last-entry rules and refitted each analysis. Test outcomes did not select these alternatives. Results and sample counts are in Tables S5–S7.

The verification runs used Python 3.12.14, NumPy 2.3.5, pandas 3.0.1, SciPy 1.18.1 and scikit-learn 1.9.0 [24,25,26]. We retained chronological split assignments, reconstructed series, model predictions and deterministic plotting scripts. A full in-memory rebuild from source-derived records reproduced the original primary forecast metrics. The Supplementary Material documents the partition rule, simple-baseline settings and sensitivity results. Raw record access remains restricted as stated below.

## 3. Results

### 3.1. Reconstruction Accuracy and Instrument Records

The retained monitoring workbooks linked 20 physical instruments and 65 anchor channels to the processing archive. Equation (1) reproduced 4209 of 4211 displayed displacements within 10^−6^ mm. The two residuals were 0.00284 mm. The reconstructed values were used downstream. Displayed values and reconstruction residuals remained separate audit fields.

The ledger matched 64 of 65 sensor identifiers. All linked conversion coefficients agreed with their workbook entries. Coefficients ranged from 0.0173 to 0.0284, with a median of 0.0178, in the workbook-reported mm kHz^−2^ convention. Three linked channels had model and range disagreements; one other identifier remained unlinked. These flags identify the records requiring specification checks before further operational use.

### 3.2. Depth-Resolved Deformation and Observation Support

**Figure 2:**
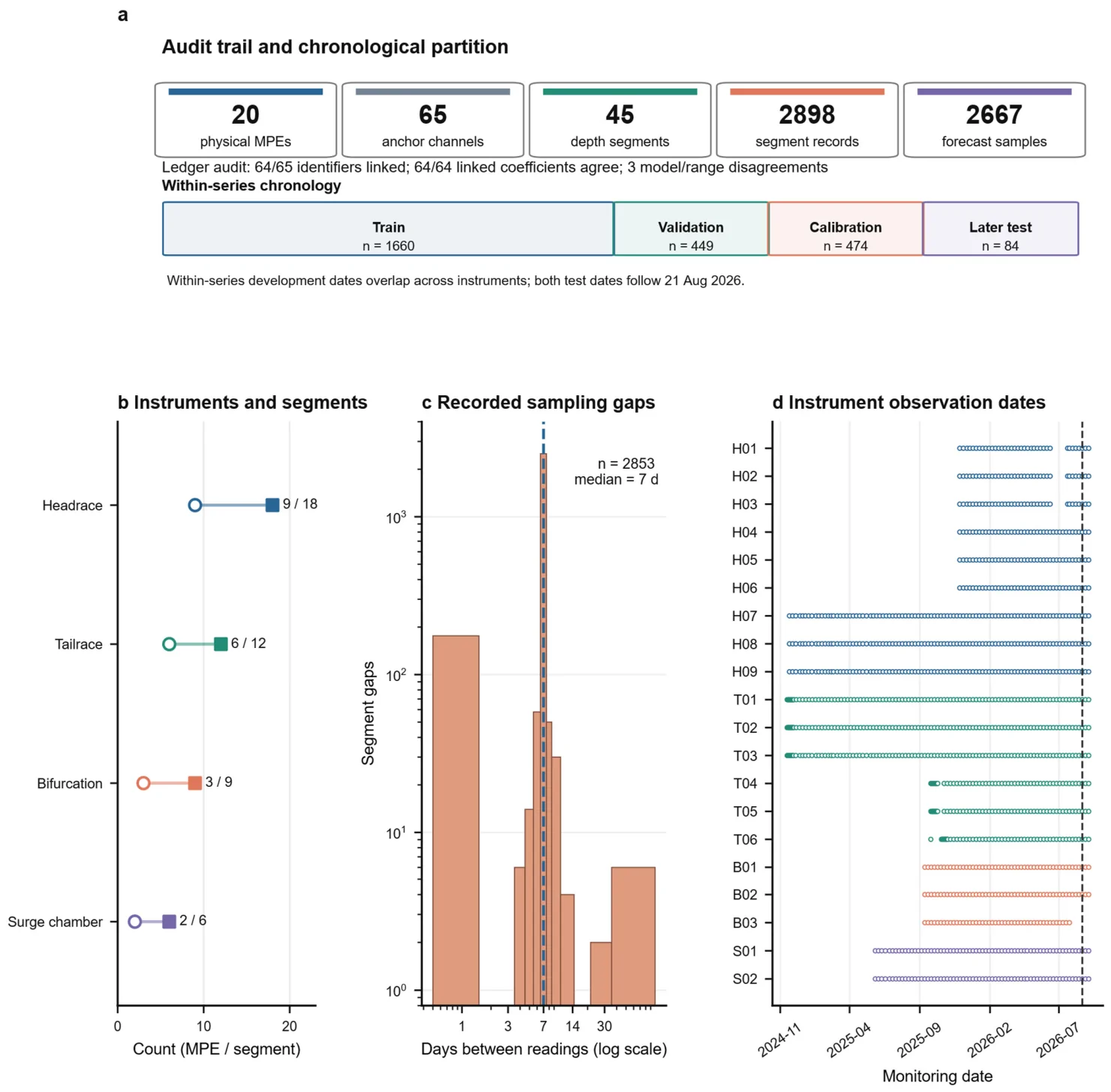
Retained data and temporal support. (**a**) Measurement hierarchy and primary per-series chronological partition. Calendar periods overlap between instruments. (**b**) Physical-instrument and adjacent-segment counts by area. (**c**) All 2853 defined positive sampling gaps on logarithmic axes; the first gap in each of the 45 series is undefined. (**d**) Recorded observation dates for 20 instruments; aliases are listed in Supplementary Table S1. The dashed line marks the analytical cutoff of 21 August 2026. Counts refer to retained records, not the complete design inventory. In (**b**), open circles denote instruments and squares denote segments. The dashed line in (**c**) marks the seven-day median.

The retained hierarchy comprised 20 instruments, 65 anchor channels, 45 adjacent-anchor segments and 2898 observations. The identity 45 = 65 − 20 holds because each consecutively ordered instrument contributes one fewer segment than the anchor channels. Figure 2 shows the counts, recorded sampling gaps and instrument-level observation support. Most gaps were seven days, but shorter and longer intervals were retained.

**Figure 3:**
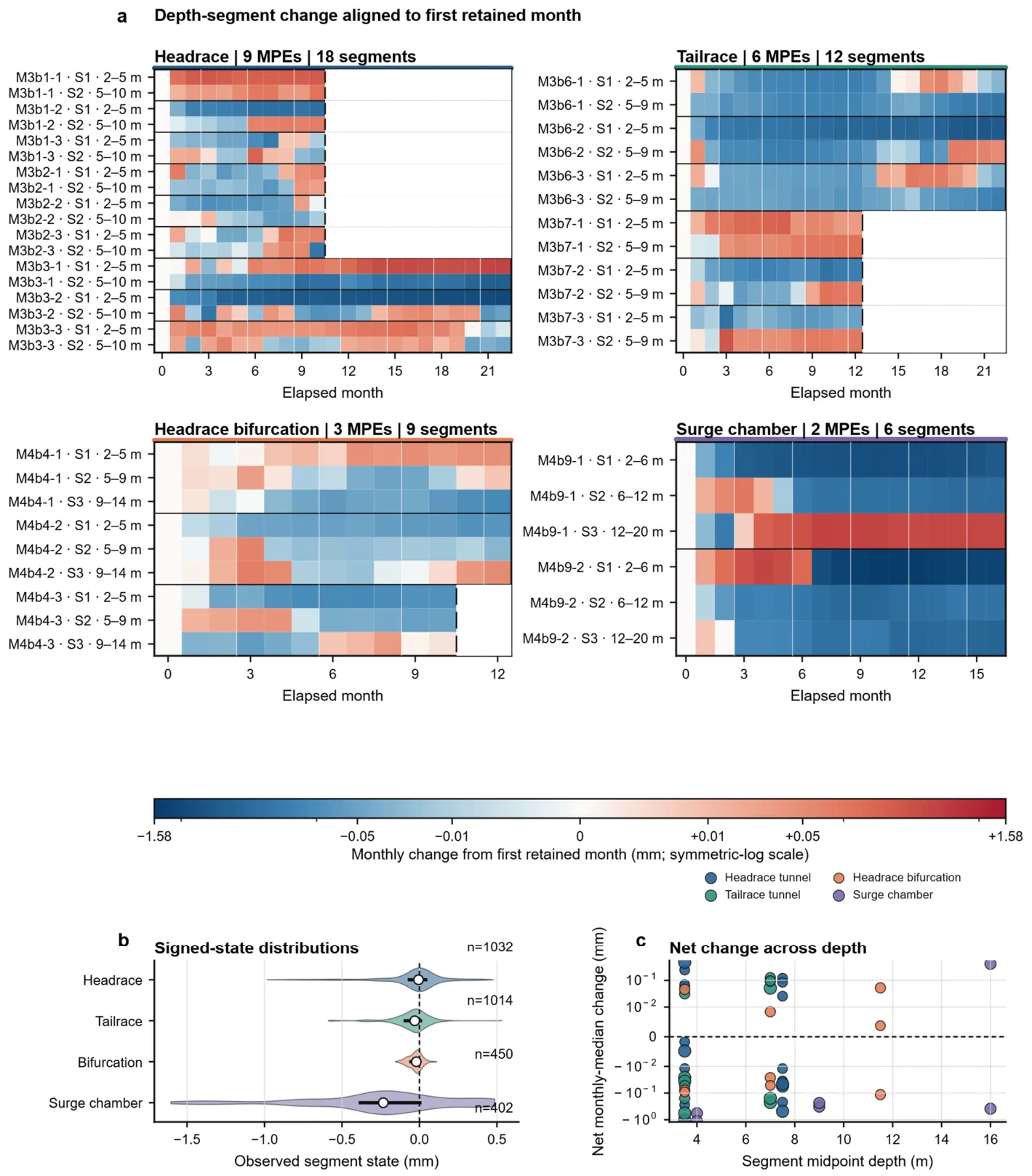
Depth-resolved evolution. (**a**) Four area matrices contain all 45 segments, grouped by instrument. Monthly medians are centred on each segment’s first retained month. Axes show elapsed months, not a common calendar. The full symmetric-log scale is linear within ±0.01 mm. Internal missing months are indicated within the retained span; space after the terminal mark lies beyond a row’s retained span. (**b**) Signed-state distributions for all 2898 observations, with Gaussian densities, median points and interquartile bars. (**c**) Last-minus-first monthly change versus midpoint depth; marker area increases with the number of observed months. No interpolation or value clipping is applied. Source instrument IDs are shown; aliases are provided in Table S1.

**Figure 4:**
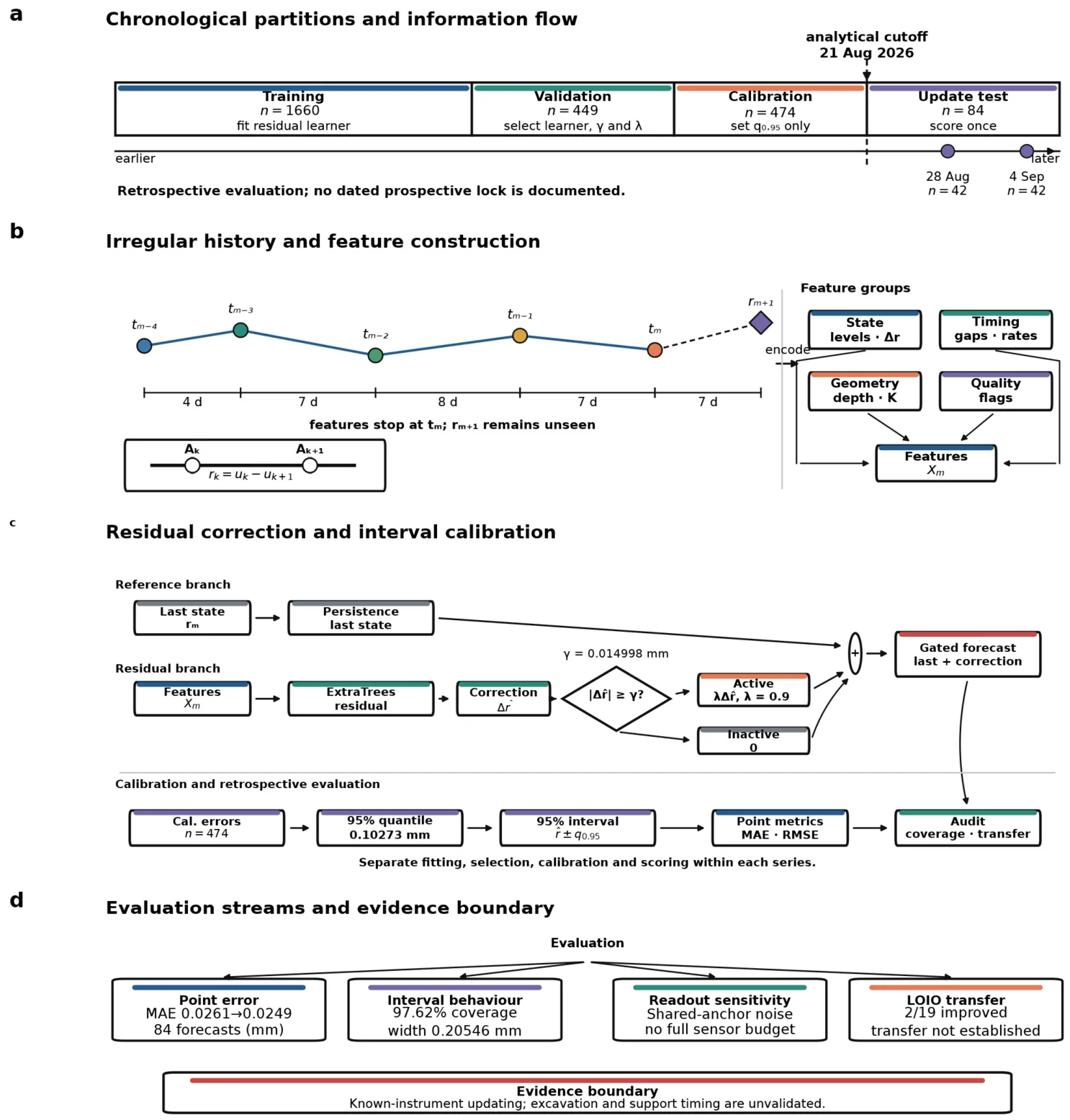
Analytical workflow. (**a**) Training, validation and calibration are chronological within each segment. Development calendar periods overlap between instruments; both test rounds follow the common cutoff. (**b**) Illustrative five-observation window and feature groups. (**c**) Persistence and residual branches, the validation-selected gate and shrinkage, and method-specific intervals calibrated on the same 474 records. The gate is active when the predicted absolute increment is at least γ. (**d**) Evaluation streams and the limits of the evidence. All input features end at the forecast origin. This retrospective design does not establish a prospective model lock. Bold text labels the steps. LOIO denotes leave-one-instrument-out refitting; MAE denotes mean absolute error and RMSE denotes root mean square error.

Figure 3a compares all 45 segment histories after aligning each series to its first retained month and subtracting that month’s median. This displays changes within each available observation span. A common symmetric-log scale preserves sign and range. Internal missing months are indicated. The space beyond the last reading lies outside a retained series. Figure 3b shows signed-state distributions, and Figure 3c relates monthly change to midpoint depth. Median absolute states were 0.0519, 0.0564, 0.0299 and 0.3059 mm for the headrace, tailrace, bifurcation and surge chamber, respectively. The observed contrast identifies records for closer review, without assigning its cause. Figure 4 summarises the forecast procedure.

### 3.3. Seven-Day Forecast Performance

On the 84 later forecasts, persistence gave MAE 0.026113 mm and RMSE 0.045921 mm. The low-dimensional lagged linear model gave MAE 0.026001 mm; exponential smoothing gave 0.027546 mm. Direct ExtraTrees residual correction had the lowest MAE and RMSE, 0.024819 and 0.042559 mm. The gated variant gave 0.024915 and 0.043989 mm, improvements of 4.59% and 4.21% over persistence (Table 5). It changed 13/84 forecasts and retained the last reading for the other 71. The result shows a small test-set gain for residual correction. Gating did not outperform the direct model. Table 6 compares the matched prediction intervals.

The training-defined high-change threshold identified 21 test samples. Their MAE decreased from 0.071302 mm under persistence to 0.066124 mm under gated correction, a 7.26% reduction. For the other 63 samples, MAE increased slightly from 0.011050 to 0.011179 mm. The aggregate benefit thus accompanied a larger error reduction in the high-change subset and a small penalty in the lower-change subset.

**Figure 5:**
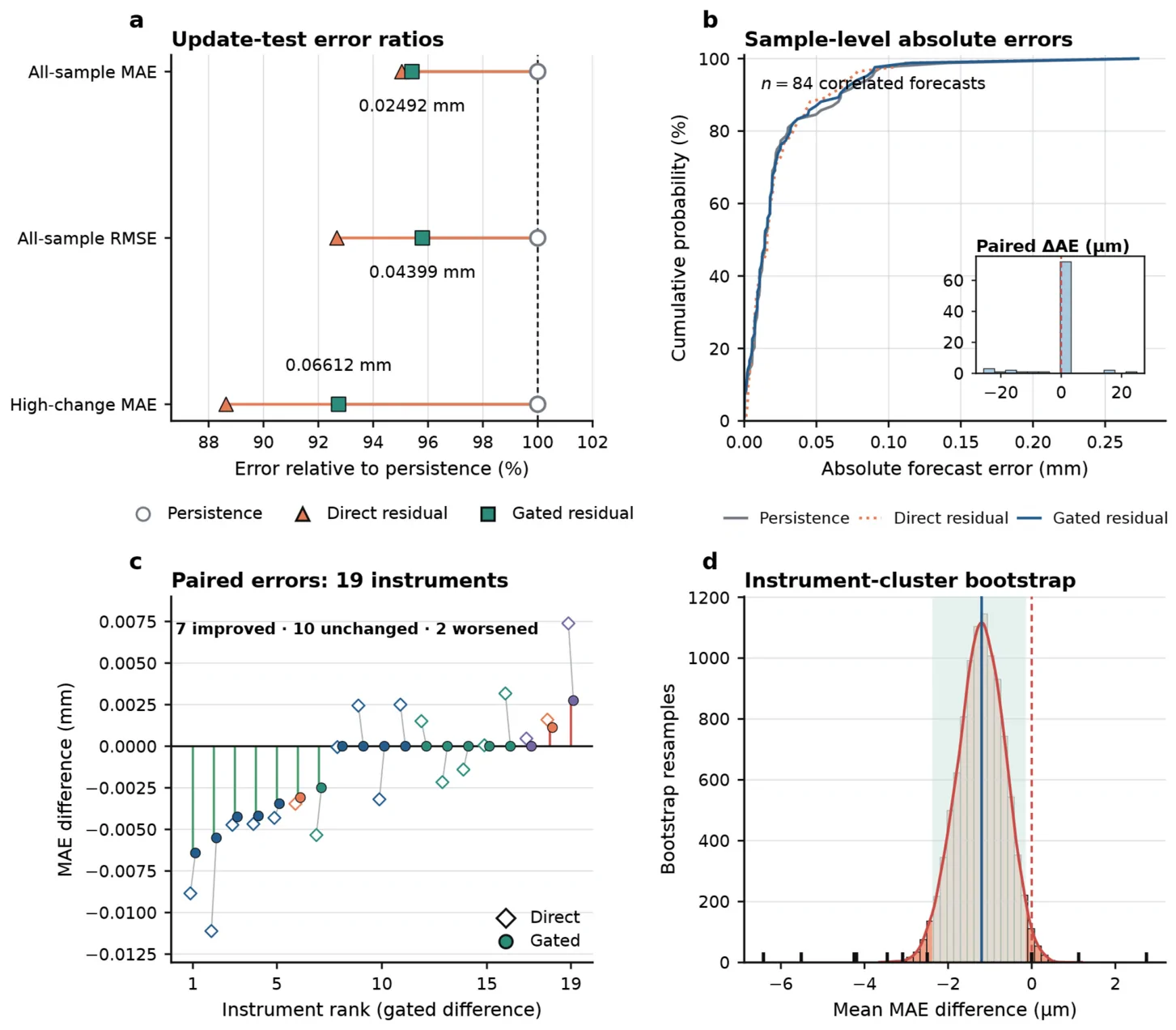
Point-error comparison. (**a**) All-sample MAE and RMSE (84 forecasts) and high-change MAE (21 forecasts), relative to persistence. (**b**) Cumulative absolute-error distributions, with individual gated-minus-persistence differences inset. (**c**) Paired MAE differences for 19 instruments; diamonds denote direct and circles gated correction. (**d**) Sample-weighted gated-minus-persistence differences from 10000 instrument-cluster resamples. The curve is a density estimate, the solid vertical line the observed difference, the shaded band the central 95% descriptive resampling interval, and the dashed vertical line zero. Shared-date dependence is not captured by this resampling. MAE denotes mean absolute error and RMSE denotes root mean square error. In (**a**), open circles, triangles and squares denote persistence, direct correction and gating.

The sample-weighted gated-minus-persistence MAE difference was −0.001198 mm. Resampling the 19 instruments as clusters gave a central 95% descriptive interval of [−0.002377, −0.000142] mm over 10,000 draws (Figure 5d). This small numerical gain is comparable to the displacement associated with one stored readout half-step when multiplied by a typical channel coefficient. It cannot be interpreted as a metrologically resolved improvement without calibrated readout, coefficient and channel-covariance uncertainties. Figure 5c also shows instruments on which correction worsened error.

### 3.4. Prediction Intervals and Calibration

**Figure 6:**
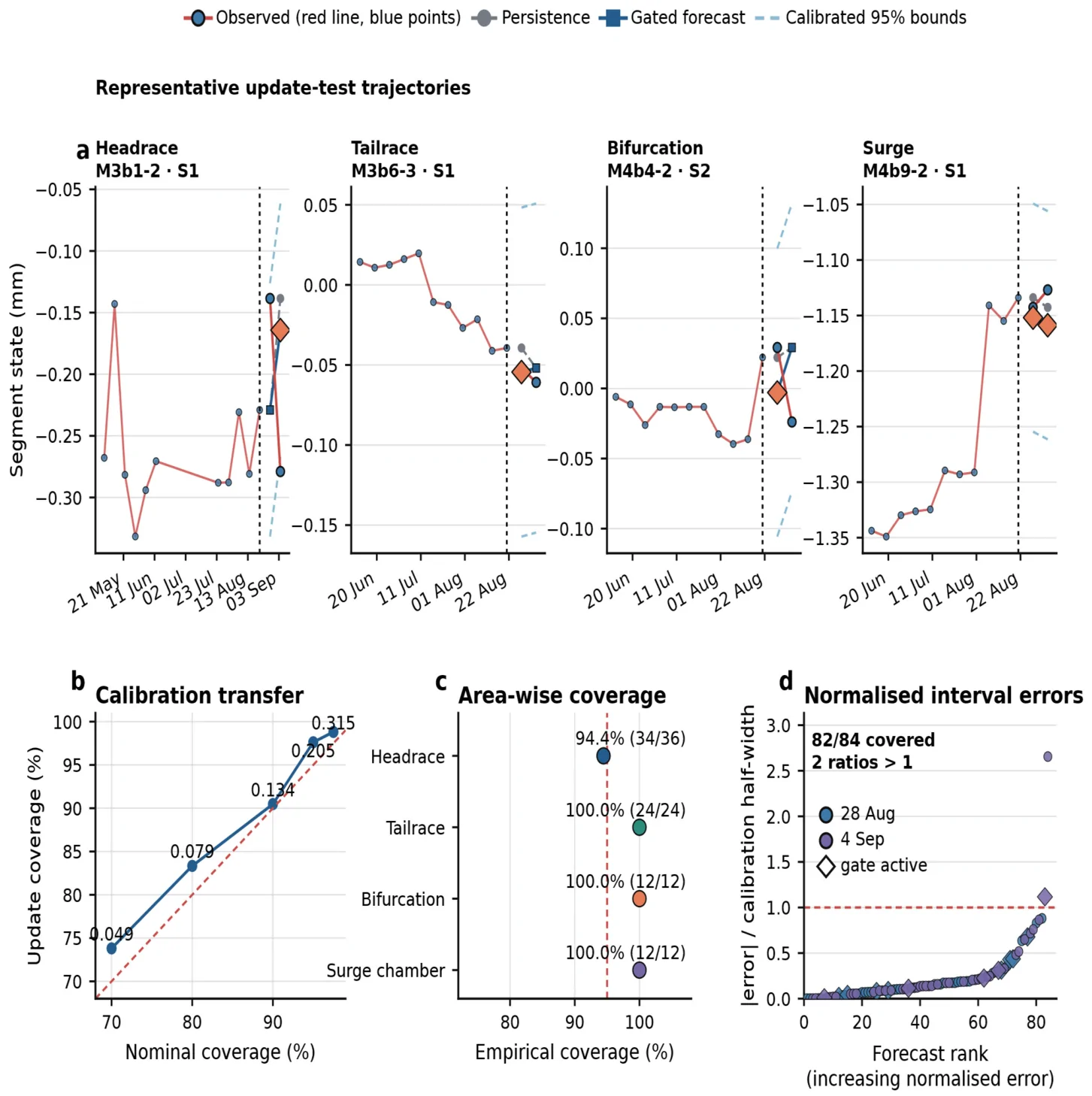
Forecast-interval assessment. (**a**) Four identified example segments with observation lines and points, persistence forecasts, and gated forecasts with nominal 95% interval bounds. Diamonds identify active gates. All plotted dates are in 2026; dashed lines mark 21 August. These examples are illustrative. (**b**) Coverage at five nominal levels using gated residuals from the calibration records; labels show full interval widths in mm. (**c**) Covered/total counts for the gated 95% interval; counts are correlated and no independent-binomial error bars are shown. (**d**) All 84 absolute errors divided by the interval half-width, ranked in ascending order. Diamonds denote active gates. Two ratios exceed one: M3b1-2 S1 and M3b2-3 S2. In (**b**), the curve shows observed coverage and the dashed line denotes equality with the nominal level. The dashed lines in (**c**,**d**) mark 95% coverage and a normalised error of one, respectively.

The gated nominal 95% interval covered 82/84 later observations (97.62%) at a full width of 0.205460 mm. Coverage was 42/42 on 28 August and 40/42 on 4 September; the area counts were 34/36 in the headrace, 24/24 in the tailrace and 12/12 in each remaining area (Figure 6c). These are observed counts, not evidence of reliable 95% coverage at a new site or date.

Persistence also covered 82/84 observations, with a wider interval of 0.229260 mm. Gating was 10.38% narrower at that equal observed coverage. Direct correction covered 81/84 with width 0.205414 mm. Its mean interval score, 0.286419 mm, was lower than gated correction (0.292174 mm) and persistence (0.316865 mm; Table 6). Direct correction is the strongest aggregate point and interval-score comparator in this dataset. Gating offers an explicit selective-update rule, which may help an engineer distinguish a retained baseline from an applied model correction, but it is not presented as the best predictor.

### 3.5. Readout Sensitivity and Instrument Transfer

**Figure 7:**
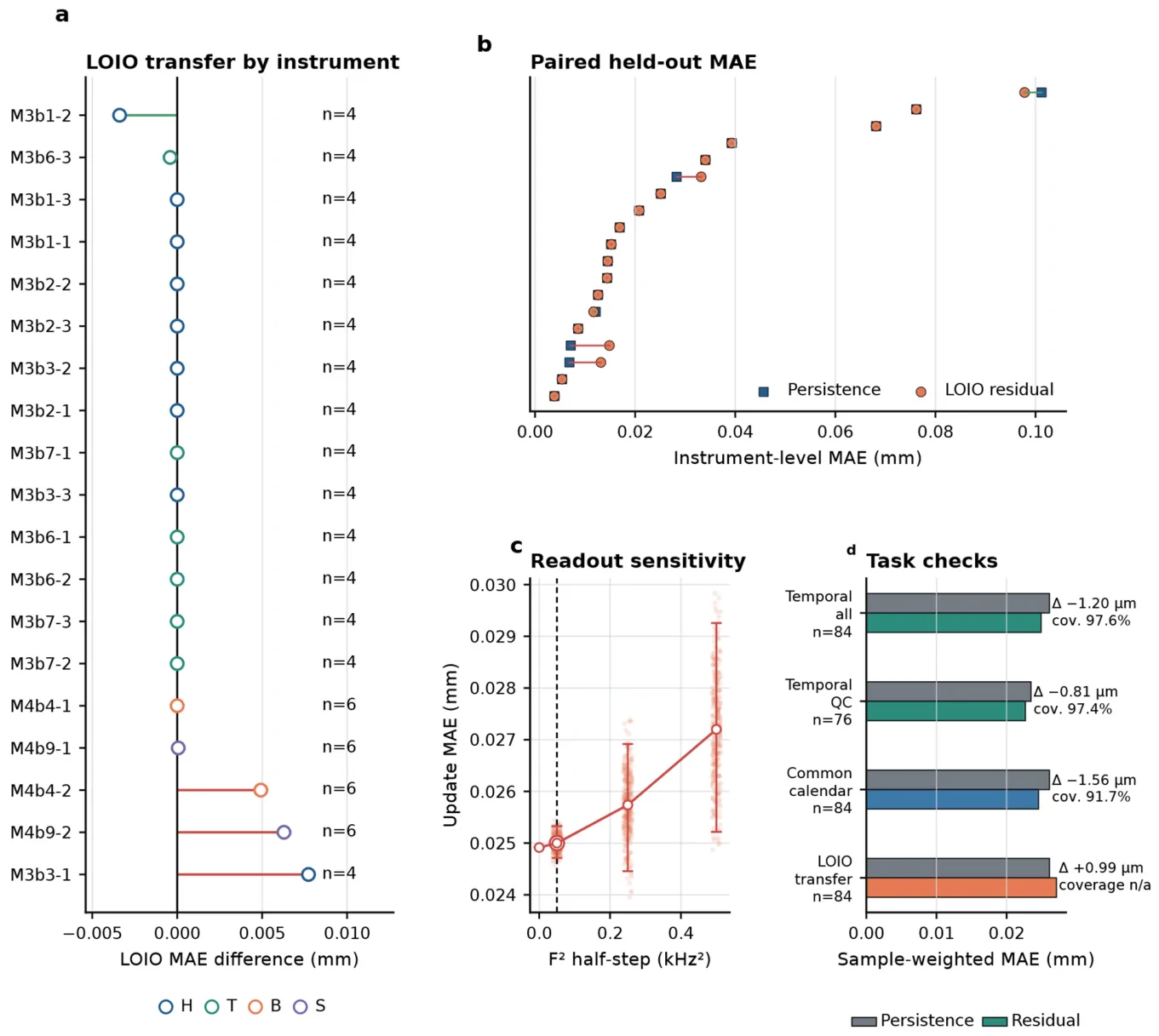
Transfer and sensitivity checks. (**a**) LOIO-refitted minus persistence MAE for all 19 held-out instruments. Globally selected settings remain fixed. (**b**) Paired held-out MAEs ranked by persistence error. (**c**) Gated MAE under shared-anchor readout perturbations. Points show 300 replicates at each non-zero level; circles and bars show means and central 95% simulation ranges. The horizontal axis gives the half-step in the source-reported kHz^2^ scale. (**d**) Primary, QC-excluded, common-calendar and LOIO point errors. Annotations give gated 95% coverage, where evaluated. The common-calendar partition is a retrospective sensitivity check, not a replacement primary analysis. LOIO denotes leave-one-instrument-out refitting and QC denotes quality control. In (**a**), H, T, B and S denote headrace, tailrace, bifurcation and surge chamber, respectively. In (**b**), squares denote persistence and circles denote LOIO correction. The dashed vertical line in (**c**) marks the primary half-step of 0.05.

At the stored readout half-step of ±0.05 in the workbook scale, shared-anchor perturbation gave a gated mean MAE of 0.025000 mm, with a central 95% simulation range of 0.024713–0.025327 mm (Figure 7c; Table S3). The unperturbed value was 0.024915 mm. At ±0.25 and ±0.50, mean MAE rose to 0.025738 and 0.027201 mm. In the three perturbation sets, gated error remained below persistence in 300/300, 300/300 and 290/300 replicates. These are results of the specified numerical perturbations; coefficient calibration and environmental effects were not simulated.

Excluding test observations associated with the unlinked identifier or model/range disagreements left 76 samples. Persistence and gated MAE were 0.023484 and 0.022671 mm, with 97.37% gated interval coverage. This restricted check retained the direction of the temporal error difference, although it cannot identify which metadata entry is correct.

LOIO refitting reduced error for 2 of 19 instruments. Its sample-weighted MAE was 0.027105 mm, compared with 0.026113 mm for persistence. The median instrument difference was zero because many gates retained their previous state (Figure 7a). This result supports validation at each instrument before transferring the fitted correction.

The common-calendar analysis selected ExtraTrees with minimum leaf size 5, *γ* = 0.017317 mm and *λ* = 1.0. Gated MAE was 0.024552 mm against 0.026113 mm for persistence, but empirical interval coverage fell to 77/84 (91.67%) at a width of 0.141360 mm (Figure 7d; Table S4). The point-error direction persisted while interval calibration depended materially on partition choice.

## 4. Discussion

### 4.1. Engineering Use of Depth-Resolved Sensor Records

Linking source readouts, coefficients and geometry to segment states makes individual discrepancies traceable. The numerical check found two workbook disagreements; register matching found three model/range discrepancies and one unmatched identifier. These are specific records to review before a channel plot is interpreted or used in forecasting. Retaining the source and reconstructed values side by side preserves the audit trail.

Adjacent-anchor states provide a consistent depth interval for reviewing three- and four-anchor instruments. The surge chamber had a median absolute state of 0.3059 mm, compared with 0.0299–0.0564 mm in the other areas. This observed contrast gives a basis for selecting records for closer review. It does not by itself rank structural safety, because the states also depend on anchor geometry and reference history. Within an instrument, comparing neighbouring segments helps distinguish a depth-specific change from a movement shared across the measured channels.

Persistence remains a practical baseline for weekly monitoring. The gated 4.59% MAE reduction corresponds to 0.001198 mm across the 84 test forecasts; the high-change subset improved by 7.26%. Direct correction performed slightly better overall. The gate’s practical role is narrower. It makes model changes selective and visible, retaining persistence for 71 forecasts. An engineer can then review the 13 altered predictions against the next readout and neighbouring intervals.

For field use, a forecast record should identify the instrument, anchor interval, latest measured state, intended next-reading date, point forecast, empirical interval and whether the gate changed persistence. A disagreement at the next reading should trigger source-readout and adjacent-segment checks before it is interpreted as new rock movement. This workflow supplies a review procedure, not a calibrated safety threshold.

### 4.2. Interpretation of Segment States and Readout Precision

The adjacent-anchor transformation is independent of the forecasting algorithm. A channel reports movement at one depth relative to the instrument reference. The difference between neighbouring channels describes relative movement within their shared interval. Localised movement can therefore appear as unequal or opposite segment changes even when the shallow cumulative channel remains smooth.

The representation also accommodates variable channel counts. A three-anchor instrument yields two segment states, while a four-anchor instrument yields three. Midpoint depth, thickness, segment index and same-instrument context provide a common description without padding every borehole to a fixed-length vector. Equivalent deformation per metre aids comparison of unequal anchor spacing, but it remains a one-dimensional interval descriptor rather than a continuum strain tensor.

Differencing changes the error structure. Independent channel noise increases the variance of *r*, whereas positively correlated common-mode errors may partly cancel. One faulty anchor affects two neighbouring segments. These relationships justify physical-instrument clustering in the inferential analysis and suggest practical quality checks based on neighbouring-segment coherence. Calibration history, temperature response, reference stability and anchor coupling should be acquired in a future deployment so that Equation (4) can be evaluated numerically.

The analysis files retain more decimal places than an operational displacement report should display. With the median coefficient of 0.0178 mm kHz^−2^, a 0.05 kHz^2^ stored readout half-step corresponds arithmetically to about 0.00089 mm for one channel. The two-channel segment and two-epoch forecast difference have additional uncertainty and possible covariance. The 0.001198 mm mean forecast gain cannot be assigned physical significance until these components are calibrated. Reported model metrics are computational quantities, not a claim of sensor resolution.

Shared-anchor perturbation at the stored half-step changed the gated MAE from 0.024915 to a mean of 0.025000 mm. This tests only one quantisation-like component under the exported convention. It cannot substitute for coefficient, temperature, reference and coupling tests. Source precision should be retained during calculations. Reporting precision should follow the verified instrument specification and uncertainty budget.

### 4.3. Calibration and Local Validation

In the main partition, the gated interval was 10.38% narrower than persistence at the same observed 82/84 coverage. Direct correction had the lowest interval score, so the gate should be justified by its selective-update behaviour rather than an accuracy claim. Method-specific residuals from the same calibration records made the width comparison matched.

The gated interval width was 8.25 times its MAE. Under the common-calendar partition, a narrower 0.141360 mm interval covered only 77/84 observations. An operational workflow should check recent calibration residuals and observed miss counts before reusing an interval after a schedule or site change. The two-date test does not establish stable nominal coverage.

LOIO refitting improved 2/19 held-out instruments and produced a sample-weighted MAE of 0.027105 mm, above persistence. For a new instrument, first verify anchor ordering, reference epoch and conversion metadata; then compare persistence and any correction on a local chronological validation block. Estimate an interval using later, separate calibration observations and record the model version before the next prospective reading. If local validation does not favour correction, retain persistence until more evidence is available.

### 4.4. Scope of the Engineering Conclusions

The test contains 84 forecasts from 19 instruments but only two acquisition dates. Segments share anchors, histories overlap and instruments experience the same field rounds. Instrument-cluster resampling retains within-instrument dependence but does not capture variation between independent dates. The point-error and coverage results describe this retrospective sample. More independent rounds are required to estimate stability through time.

The records support depth-specific review and next-observation forecasting, but they do not separate instrument drift from excavation, support or groundwater response. Future attribution needs timestamped heading positions, advance and support records, and groundwater and temperature series, along with independent reference checks. These inputs were unavailable here and were not reconstructed from the deformation series.

Before operational transfer, each location needs checked sensor specifications and reference conventions, a local chronological comparison with persistence, a separate residual-calibration period and prospective recording of the next scheduled readings. Model and calibration versions should be locked before each prospective test. The present dataset cannot support a transferable safety-warning threshold.

### 4.5. Establishing Measurement Traceability in a Future Deployment

A field uncertainty budget should begin with the manufacturer’s calibration certificates for readout frequency and channel coefficient, their validity dates and stated units. At installation, the record must include anchor depths, borehole orientation, coupling and the reference epoch. During monitoring, log temperature compensation, acquisition-clock checks, sensor replacement and independent reference observations. The conversion and adjacent-channel covariance in Equation (4) can then be evaluated with stated standard uncertainties and repeatability tests. This would permit a calibrated statement about relative segment movement; the workbook reconstruction in this study alone cannot do so.

Prospective forecast evaluation should be kept separate from that measurement budget. Lock the conversion convention, model and calibration set before each new monitoring round. Report measured segment states with calibrated uncertainty alongside the point forecast and empirical predictive interval. When source readouts or neighbouring segments disagree, investigate the instrument record before attributing the change to excavation or support. Timestamped construction, groundwater and temperature records are needed for physical attribution.

## 5. Conclusions

We assessed a source-linked, depth-resolved review and next-reading forecasting workflow for irregular multipoint-extensometer records in a pumped-storage water-conveyance system. The findings are:

1. Source-linked conversion reproduced 4209 of 4211 displayed values within 10^−6^ mm and exposed two arithmetic discrepancies. Ledger matching found three model/range disagreements and one unmatched sensor. These checks locate records that require instrument-level review; they do not independently calibrate displacement.
2. The 65 anchor channels formed 45 adjacent-anchor segments across 20 instruments. The segment representation gives each observation and forecast a defined depth interval and accommodates three- and four-anchor instruments. Median absolute states differed between areas, with the largest value, 0.3059 mm, in the surge chamber.
3. On 84 later seven-day forecasts, direct residual correction had the lowest MAE, 0.024819 mm. Gating changed 13 forecasts and reduced persistence MAE from 0.026113 to 0.024915 mm, a 0.001198 mm or 4.59% difference. A simple lagged linear baseline gave 0.026001 mm. The numerical gain is too small to claim a physically resolved improvement without a complete uncertainty budget.
4. The gated nominal 95% interval was 10.38% narrower than persistence at the same 82/84 observed coverage, but common-calendar calibration covered 77/84. These empirical counts support local interval checking, not an exact 95% coverage claim.
5. Readout half-step perturbation gave a gated mean MAE of 0.025000 mm; held-out-instrument refitting improved 2/19 instruments. Duplicate handling and history length also changed the fitted gate and test error (Tables S5–S7). Local validation and source-level quality checks are therefore necessary before using corrections elsewhere.

The supported engineering use is a traceable review of measured depth intervals and a checkable forecast for the next reading. Attribution of movement and operational warning thresholds require independent field and metrological evidence.

## Figures and Tables

**Table 1:** Monitoring data retained for adjacent-anchor segment analysis.

Area	Physical MPEs	Segments	Segment Observations	Observation Period	Median |State| (mm)
Headrace tunnel	9	18	1032	21 November 2024–4 September 2026	0.0519
Tailrace tunnel	6	12	1014	16 November 2024–4 September 2026	0.0564
Headrace bifurcation	3	9	450	12 September 2025–4 September 2026	0.0299
Headrace surge chamber	2	6	402	27 May 2025–4 September 2026	0.3059

Note. MPE, multipoint extensometer. Segment observations are the retained adjacent-anchor states before forecast-sample construction.

**Table 2:** Measurement chain, audit evidence and uncertainty boundary.

Quantity	Operational Definition	Principal Uncertainty Sources	Treatment and Boundary
Stored sensor readout *F*^2^	Exported squared-frequency readout; workbook label kHz^2^, retained step 0.1	Readout quantisation, acquisition and export precision	Used with channel-specific *K*; half-step perturbation tested separately.
Anchor reading *u_i_*_,*k*_	Displacement reconstructed by Equation (1)	Calibration, sensitivity coefficient, reference stability, temperature and coupling	4209/4211 displayed values reproduced within 10^−6^ mm; two discrepancies retained.
Register metadata	Sensor ID, *K*, model and nominal range	Transcription, stale register and identifier mismatch	64/65 IDs linked; all linked *K* values matched; three model/range mismatches.
Segment state *r_i_*_,*k*_	Difference between adjacent anchor readings, Equation (2)	Both channel uncertainties and their correlation	Propagation model stated in Equation (4). Shared and independent error components are distinguished conceptually.
Temporal change and rate	Difference between segment states at two epochs, divided by actual elapsed time where required	Two-epoch measurement error, timestamp precision and short-interval amplification	Actual timestamps retained; robust trend and gap descriptors used.
Point forecast	Next valid observation of *r_i_*_,*k*_	Model error, sampling irregularity, regime change and omitted construction variables	Evaluated on a later chronological block against persistence.
Empirical 95% prediction interval	Calibration-residual band around the gated point forecast	Finite calibration sample, serial dependence and area heterogeneity	Reported as predictive uncertainty only; no exact conditional-coverage or metrological-uncertainty claim.

Note. *u*_c_ denotes combined standard uncertainty. The distinction between measurement uncertainty and forecast uncertainty is retained throughout the analysis.

**Table 3:** Feature groups supplied to the residual learner.

Feature Group	Variables	Leakage-Control Rule
State history	Last value, four preceding lags, latest and preceding increments	Five observed values only
Time-aware dynamics	Latest and preceding velocities, robust pairwise slope, acceleration proxy	Actual day intervals
Local variability	Rolling mean, standard deviation and range	Historical window only
Sampling pattern	Forecast horizon, mean gap, gap coefficient of variation, history-window duration	Natural time
Segment geometry and metadata	Midpoint depth, thickness, segment index and mean K	Static or pre-recorded attributes
Instrument context	Total shallow-to-deep movement; cross-segment mean, standard deviation and maximum absolute state	Historical endpoint only
Engineering identifiers	Area, installation position, chainage and chainage-availability flag	Physical instrument ID excluded
Quality indicators	Duplicate-time, long-gap and conversion-discrepancy flags within history	Not imputed

**Table 4:** Validation-only comparison of full-feature learners and simple statistical baselines (*n* = 449).

Learner	Setting	MAE (mm)	RMSE (mm)	High-Change MAE (mm)	Objective
ExtraTrees	minimum leaf = 10	0.020305	0.034585	0.056319	0.048465
ExtraTrees	minimum leaf = 5	0.020543	0.034620	0.056135	0.048611
Random forest	minimum leaf = 8	0.020696	0.034654	0.055835	0.048613
Ridge regression	*α* = 1000	0.020033	0.034762	0.057657	0.048862
Ridge regression	*α* = 100	0.020906	0.035400	0.058043	0.049927
Histogram gradient boosting	15 terminal nodes	0.022327	0.035984	0.056009	0.050332
Ridge regression	*α* = 10	0.021537	0.035843	0.058348	0.050712
Persistence	last measured state	0.019326	0.034270	0.057086	0.047869
Lagged linear	ridge α = 1000; six features	0.019860	0.034468	0.057268	0.048494
Exponential velocity smoothing	*α* = 0.2; scale = 0.25	0.019768	0.034947	0.058292	0.048914

Note. Objective = overall MAE + 0.5 × high-change MAE. The low-dimensional linear model uses the last two increments, their rates, a robust historical slope and the forecast horizon. The exponential smoother weights the four historical rates; its scale controls extrapolation. Persistence has no fitted parameter. All settings were selected without access to the later test dates. MAE, mean absolute error; RMSE, root mean square error.

**Table 5:** Chronological seven-day point-forecast performance (84 correlated forecasts).

Method	MAE (mm)	RMSE (mm)	Bias (mm)	Gate Rate
Persistence	0.026113	0.045921	0.003086	NA
Robust trend	0.031315	0.050714	0.004104	NA
ExtraTrees residual	0.024819	0.042559	−0.000734	NA
Magnitude-gated residual	0.024915	0.043989	0.000734	15.48%
Lagged linear	0.026001	0.045680	0.000633	NA
Exponential velocity smoothing	0.027546	0.047596	0.003392	NA

Note. Bias is prediction minus observation. The linear and smoothing settings were selected on validation only. Gate activation was 13/84. Matched interval results for persistence, direct correction and gating are in Table 6. NA, not applicable.

**Table 6:** Matched nominal 95% intervals on the same 84 test observations.

Method	Covered/Total	Width (mm)	Mean Interval Score (mm)
Persistence	82/84	0.229260	0.316865
Direct residual	81/84	0.205414	0.286419
Gated residual	82/84	0.205460	0.292174

Note. Each method uses its own absolute residuals from the same 474 calibration samples. Coverage is empirical on 84 correlated test cases; no exact 95% guarantee or independent-binomial interval is implied.

## Data Availability

The analysis code and non-sensitive aggregate results accompany this article as Supplementary Files. Raw monitoring records and record-level derivatives remain under project-owner access control because they contain infrastructure information. Requests for verification access can be directed to the corresponding author and require written project-owner permission.

## Supplementary Materials

The following supporting information can be downloaded at https://www.mdpi.com/article/10.3390/s26216801/s1, Table S1. Instrument aliases and adjacent-anchor intervals; Table S2. Worked reconstruction for M3b1-1; Table S3. Shared-anchor readout perturbation results; Table S4. Primary and retrospective common-calendar sensitivity; Table S5. Simple baselines and local gate sensitivity; Table S6. Five-, seven- and nine-observation history sensitivity; Table S7. Duplicate-record rule sensitivity. The Supplementary Files also contain Analysis_Code.py, Audit_Code.py and Aggregate_Results.json.

## References

[1] Zhou H., Qu C.-K., Hu D.-W., Zhang C.-Q., Azhar M.U., Shen Z., Chen J. (2017). In situ monitoring of tunnel deformation evolutions from auxiliary tunnel in deep mine. Eng. Geol..

[2] Liu D., Zhang D., Fang Q., Sun Z., Luo J., Li A. (2020). Field monitoring of the deformation and internal forces of the surrounding rock and support structures in the construction of a super-span high-speed railway tunnel: A case study. Appl. Sci..

[3] Zhao Y.-M., Han Y., Kou Y.-Y., Li L., Du J.-H. (2021). Three-dimensional, real-time, and intelligent data acquisition of large deformation in deep tunnels. Adv. Civ. Eng..

[4] Zhang X., Zhu H.-H., Jiang X., Broere W., Long L. (2024). Designing a distributed sensing network for structural health monitoring of concrete tunnels: A case study. Struct. Control Health Monit..

[5] Adoko A.-C., Jiao Y.-Y., Wu L., Wang H., Wang Z.-H. (2013). Predicting tunnel convergence using multivariate adaptive regression spline and artificial neural network. Tunn. Undergr. Space Technol..

[6] He Y., Chen Q. (2023). Construction and application of LSTM-based prediction model for tunnel surrounding rock deformation. Sustainability.

[7] Li Z., Ma E., Lai J., Su X. (2024). Tunnel deformation prediction during construction: An explainable hybrid model considering temporal and static factors. Comput. Struct..

[8] Sun J., Fang Y., Luo H., Yao Z., Xiang L., Wang J., Wang Y., Jiang Y. (2025). Hybrid deep learning approach for rock tunnel deformation prediction based on spatio-temporal patterns. Undergr. Space.

[9] Chen Y., Shao X., Chang X., Jusoh S.N.B., Lu Z., Bao H.Q., Han X., Xu J. (2025). Prediction of surrounding rock deformation in a highway tunnel using an LSTM-RF hybrid model. J. Eng..

[10] Ansari A.F., Heng A., Lim A., Soh H. Neural continuous-discrete state space models for irregularly-sampled time series. Proceedings of the 40th International Conference on Machine Learning.

[11] Schirmer M., Eltayeb M., Lessmann S., Rudolph M. Modeling irregular time series with continuous recurrent units. Proceedings of the 39th International Conference on Machine Learning.

[12] Hyndman R.J., Koehler A.B. (2006). Another look at measures of forecast accuracy. Int. J. Forecast..

[13] Tashman L.J. (2000). Out-of-sample tests of forecasting accuracy: An analysis and review. Int. J. Forecast..

[14] Bergmeir C., Benítez J.M. (2012). On the use of cross-validation for time series predictor evaluation. Inf. Sci..

[15] Bergmeir C., Hyndman R.J., Koo B. (2018). A note on the validity of cross-validation for evaluating autoregressive time series prediction. Comput. Stat. Data Anal..

[16] Cawley G.C., Talbot N.L.C. (2010). On over-fitting in model selection and subsequent selection bias in performance evaluation. J. Mach. Learn. Res..

[17] Angelopoulos A.N., Bates S. (2023). Conformal prediction: A gentle introduction. Found. Trends Mach. Learn..

[18] Stankevičiūtė K., Alaa A.M., van der Schaar M. (2021). Conformal time-series forecasting. Adv. Neural Inf. Process. Syst..

[19] Xu C., Xie Y. Conformal prediction interval for dynamic time-series. Proceedings of the 38th International Conference on Machine Learning.

[20] Oliveira R.I., Orenstein P., Ramos T., Romano J.V. (2024). Split conformal prediction and non-exchangeable data. J. Mach. Learn. Res..

[21] Joint Committee for Guides in Metrology (2008). Evaluation of Measurement Data—Guide to the Expression of Uncertainty in Measurement.

[22] Breiman L. (2001). Random forests. Mach. Learn..

[23] Geurts P., Ernst D., Wehenkel L. (2006). Extremely randomized trees. Mach. Learn..

[24] Harris C.R., Millman K.J., van der Walt S.J., Gommers R., Virtanen P., Cournapeau D., Wieser E., Taylor J., Berg S., Smith N.J. (2020). Array programming with NumPy. Nature.

[25] McKinney W. Data structures for statistical computing in Python. Proceedings of the 9th Python in Science Conference.

[26] Pedregosa F., Varoquaux G., Gramfort A., Michel V., Thirion B., Grisel O., Blondel M., Prettenhofer P., Weiss R., Dubourg V. (2011). Scikit-learn: Machine learning in Python. J. Mach. Learn. Res..

