# Supplementary material for: Depth-Resolved Deformation Monitoring and Short-Term Forecasting with Multipoint Extensometers in Hydraulic Tunnels
Source: Sensors (Basel). 2026 Oct 28;26(21):6801. doi: 10.3390/s26216801 (PMC13765777; doi:10.3390/s26216801)
Supplement: Supplementary file 1 [file sensors-26-06801-s001.zip › sensors-4615766-supplementary.pdf]

Supplementary material

Depth-Resolved Deformation Monitoring and Short-Term Forecasting with Multipoint Extensometers in Hydraulic Tunnels

Description: Instrument aliases, readout reconstruction, shared-anchor perturbations, chronological-partition sensitivity, simple baselines, gate and history-window sensitivity, and duplicate-rule checks.

Table S1. Instrument aliases and adjacent-anchor intervals.

Alias	Source ID	Area	Anchor interval (m)
H01	M3b1-1	Headrace	S1: 2–5; S2: 5–10
H02	M3b1-2	Headrace	S1: 2–5; S2: 5–10
H03	M3b1-3	Headrace	S1: 2–5; S2: 5–10
H04	M3b2-1	Headrace	S1: 2–5; S2: 5–10
H05	M3b2-2	Headrace	S1: 2–5; S2: 5–10
H06	M3b2-3	Headrace	S1: 2–5; S2: 5–10
H07	M3b3-1	Headrace	S1: 2–5; S2: 5–10
H08	M3b3-2	Headrace	S1: 2–5; S2: 5–10
H09	M3b3-3	Headrace	S1: 2–5; S2: 5–10
T01	M3b6-1	Tailrace	S1: 2–5; S2: 5–9
T02	M3b6-2	Tailrace	S1: 2–5; S2: 5–9
T03	M3b6-3	Tailrace	S1: 2–5; S2: 5–9
T04	M3b7-1	Tailrace	S1: 2–5; S2: 5–9
T05	M3b7-2	Tailrace	S1: 2–5; S2: 5–9
T06	M3b7-3	Tailrace	S1: 2–5; S2: 5–9
B01	M4b4-1	Bifurcation	S1: 2–5; S2: 5–9; S3: 9–14
B02	M4b4-2	Bifurcation	S1: 2–5; S2: 5–9; S3: 9–14
B03	M4b4-3	Bifurcation	S1: 2–5; S2: 5–9; S3: 9–14
S01	M4b9-1	Surge chamber	S1: 2–6; S2: 6–12; S3: 12–20
S02	M4b9-2	Surge chamber	S1: 2–6; S2: 6–12; S3: 12–20

Note. S1 is the shallowest adjacent-anchor interval. The borehole collar to the first anchor is not included as a segment.

Table S2. Worked reconstruction for M3b1-1.

Input or quantity	Anchor 1	Anchor 2
Depth (m)	2	5
Retained baseline date	27 November 2025	27 November 2025
Baseline readout	3159.2	3462.0
Conversion coefficient K	0.0181	0.0181
Readout on 4 September 2026	3165.8	3455.1
Reconstructed displacement (mm)	0.11946	−0.12489

Note. The source workbook labels the readout scale kHz^2 and K in mm kHz^{-2}. The adjacent-segment state is $0.11946 - (-0.12489) = 0.24435$ mm. This is an arithmetic check within the exported convention, not an independent calibration.

Table S3. Shared-anchor readout perturbation results.

Half-step	Method	Mean MAE (mm)	Central 95% simulation range (mm)
0.00	Direct residual	0.024819	0.024819–0.024819
0.00	Gated residual	0.024915	0.024915–0.024915
0.00	Persistence	0.026113	0.026113–0.026113
0.05	Direct residual	0.024815	0.024561–0.025041
0.05	Gated residual	0.025000	0.024713–0.025327
0.05	Persistence	0.026160	0.025915–0.026423
0.25	Direct residual	0.025280	0.024189–0.026317
0.25	Gated residual	0.025738	0.024457–0.026917
0.25	Persistence	0.026630	0.025375–0.027731
0.50	Direct residual	0.026598	0.024679–0.028606
0.50	Gated residual	0.027201	0.025217–0.029257
0.50	Persistence	0.027997	0.026264–0.030281

Note. 300 replicates per non-zero level; 0 denotes the unperturbed calculation. Methods share the same source-level perturbations. Half-step units follow the reported workbook scale.

Table S4. Primary and retrospective common-calendar sensitivity.

Quantity	Primary per-series	Common calendar
Training / validation / calibration	1660 / 449 / 474	1860 / 393 / 330
Test forecasts	84	84
Minimum leaf size	10	5
Gate threshold (mm)	0.014998	0.017317
Shrinkage	0.9	1.0
Persistence MAE (mm)	0.026113	0.026113
Direct residual MAE (mm)	0.024819	0.024940
Gated residual MAE (mm)	0.024915	0.024552
Gated full interval width (mm)	0.205460	0.141360
Gated coverage	82/84 (97.62%)	77/84 (91.67%)

Note. Common-calendar training ended on 30 April 2026, validation covered May–June, and calibration covered 1 July–21 August. This sensitivity was specified during reanalysis, not prospectively.

Table S5. Simple baselines and local gate sensitivity.

Analysis	Setting	Validation objective	Test MAE (mm)	Notes
Persistence	last state	0.047869	0.026113	84 test forecasts
Lagged linear	$\alpha = 1000$	0.048494	0.026001	train fit; validation selection
Exponential velocity smoothing	$\alpha = 0.2$; scale = 0.25	0.048914	0.027546	four historical rates
Primary gate	$\gamma = 0.014998$ mm; $\lambda = 0.9$	selected on validation	0.024915	13/84 corrections
Local gate range	$\gamma \times \{0.8, 1, 1.2\}$; $\lambda \in \{0.7, 0.9, 1\}$	post hoc	0.024844–0.025381	8–18 corrections

Note. The gate range shows all nine fixed-setting combinations on the same 84 test forecasts. It is descriptive, and its minimum was not used to reselect the submitted model. The simple-baseline settings were selected on validation only.

Table S6. Five-, seven- and nine-observation history sensitivity.

History	Train	Validation	Test	γ (mm)	Direct MAE	Gated MAE
5	1660	449	84	0.014998	0.024819	0.024915
7	1595	441	84	0.014742	0.024384	0.024721
9	1542	420	84	0.012321	0.025125	0.025179

Note. The histories were rebuilt from source-derived segment states in memory. A longer required history excludes some early development targets; the test targets remain the same. The fitted model family and leaf size were fixed, while γ and λ were selected afresh within each validation block. This is a post hoc robustness analysis.

Table S7. Duplicate-record rule sensitivity.

Duplicate rule	Retained raw rows	Changed segment states	γ (mm)	Direct MAE	Gated MAE
Mean of pair (primary)	4211	0	0.014998	0.024819	0.024915
First source row	4205	4	0.021363	0.024945	0.025255
Last source row	4205	4	0.022688	0.025017	0.025260

Note. Six duplicated anchor–timestamp pairs occurred at one physical instrument on 24 and 31 October 2025. First/last alternatives changed four segment states; the largest absolute state change relative to pair averaging was 0.135805 mm. Each alternative was rebuilt and refitted, with model and gate selection confined to development data. All three test sets contain the same 84 targets. The conflicting source rows are retained in the restricted audit archive; none was corrected on the basis of test error.
